# An insight into market and non-market alternative food networks in Czechia during Covid-19 and beyond

**DOI:** 10.3389/fnut.2024.1327308

**Published:** 2024-02-06

**Authors:** Zdeňka Smutná, Jan Vávra, Barbora Duží

**Affiliations:** ^1^Department of Geography, Faculty of Science, Jan Evangelista Purkyně University in Ústí nad Labem, Ústí nad Labem, Czechia; ^2^Department of Local and Regional Studies, Institute of Sociology of the Czech Academy of Sciences, Prague, Czechia; ^3^Department of Environmental Geography, Institute of Geonics of the Czech Academy of Sciences, Brno, Czechia

**Keywords:** Agri-food value chains, alternative food networks, COVID-19, food self-provisioning, food behavior, resilience

## Abstract

**Introduction:**

The Covid-19 pandemic affected food systems in many countries and emphasized a lot of already existing social, economic and environmental agri-food problems. Alternative food networks (AFNs), praised for their ability to improve the food systems, were under stress, however, at the same time, the changed conditions may have opened new possibilities. In this paper we address the importance of AFNs during the pandemic and investigate how households have changed their participation in AFNs. Our research is novel by simultaneously focusing on both market and non-market AFNs which are often studied separately.

**Methods:**

A representative questionnaire study of Czech households was carried out in Autumn 2021 to provide a case study of food and consumption behavior of the European country after several waves of Covid-19.

**Results and discussion:**

Based on the responses of 515 participants, the results show that 68% of Czech households participate in some form of AFNs, be it shopping or food self-provisioning, i.e., non-market food procurement in the form of gardening. Focusing on the market AFNs, farmers’ markets and farm gate sales are the most popular. Covid-19 and 2021 emerging economic pressures led to a decrease of consumption of organic food (22% of respondents) as well as fresh fruit and vegetables in general (10% of respondents) and a noticeable occurrence of food insecurity (18% of households). Based on these findings, the paper discusses the ability of AFNs to support food and nutritional resilience. Problems which may endanger market-oriented AFNs are discussed as well.

**Conclusion:**

By addressing both market and non-market AFNs, the paper brings new knowledge into the food environment and agri-food policies research.

## 1 Introduction

In light of the Covid-19 pandemic and subsequent socio-economic changes, weaknesses and problems were exposed uncompromisingly in the entire food chain. It disclosed high dependence on just-in-time supplies leading to short-run disruptions during unexpected events (1). Furthermore, on the supply side–producers, suppliers, and retailers across the chain–all had to deal with issues such as employee cuts, stricter hygiene rules or other government anti-pandemic measures (2, 3). Many of them had to face a loss of market demand caused for instance by closures of restaurants, bans on farmers’ markets and festivals, etc. Applied measures also affected the consumer side, such as various mobility restrictions or lockdowns affecting their demand, frequency, and amount of shopping (4, 5). Moreover, on the consumer side, the effects of the pandemic are addressed in a number of studies that highlight concerns about adopting unhealthy eating habits combined with psychological problems (6, 7). In addition, the problem of unavailability of quality and fresh food due to financial problems may have deepened (6, 8), as evidenced by the growing increase in the interest in services of food banks (9, 10). To sum up, these disruptions in food supply during the pandemic and associated socio-economic changes have tested the resilience and adaptability of each segment within the food supply chain (4, 11).

In response to emerging issues of the globalized agri-food system during the pandemic, international communities called for actions to prevent disruption of food supply chains. The Food and Agricultural Organization of the United Nations (FAO) points out that mainly small and medium agribusiness enterprises are at risk due to pandemic (12). Among others, FAO calls for building local food production to build more resilient local food systems (13) and a set of public financial support and policy responses (14, 15). Furthermore, re-localization trends are receiving more attention and researchers are calling attention to the need for transformation – the development of environmentally friendly practices and the promotion of short food supply chains (16–18). Some authors see the pandemic as an opportunity for a more dynamic process of converging consumers with food production (1, 19), and communities are seen as the most important drivers of change (16, 20, 21).

Given the aforementioned themes and research results, this paper contributes to the discussion about the trends observed among consumers, which can support or, on the contrary, threaten the transformation of the agri-food system with regard to local food orientation and sustainability. Following this, it is crucial to capture information about consumer practices in relation to alternative food networks (AFNs). In this study, we perceive the AFNs concept as a framework referring to connecting producers with consumers as well as to sustainable practices within food production and consumption (22). The value-added of our research is the inclusion of AFNs operating outside the market–namely food self-provisioning (further as FSP), considering it an important and widespread element of a non-commercial way of obtaining food, which can play an important role in social resilience (23), namely in rural areas (24). Both AFNs and FSP have strong potential to contribute to the sustainable, even post-growth, transformation of food systems and can be seen as already existing alternatives to the dominant globalized food chains (25).

This study is inspired by the research focused on the impact of the pandemic on the whole agri-food system, including wider food policy implications for Czechia as a mid-sized post-socialist country with specific agricultural and food systems situated in Central Europe. In this paper, food consumption is placed in the context of the whole agri-food system and the findings presented here are key to the discussion of sustainability and resilience as well as the changes that can enhance or threaten these aspects in food production and consumption. While a number of articles were based on data collected during the first wave of the pandemic in Spring 2020 [e.g., Millard et al. (5)], our study focuses on food-related behavior and local food supply using the data collected in the Autumn 2021. This enables us to gain a better overview about aspects which either became the “new normal” or returned back to the previous state after the first Covid-19 shock in 2020. The main research questions are:

*To what extent do consumers use alternative food networks to acquire food?*

*How have food acquisition and eating habits changed in the context of the pandemic?*

With regard to the above questions, our objective is to discuss some key challenges and implications for food policy.

## 2 Evidence of selected changes and trends in response to the Covid-19 pandemic

A significant number of studies have been published on changes in consumer behavior and lifestyle as a consequence of the pandemic, however, the prevalent proportion of studies reflects only the first wave of the pandemic (Spring 2020). The results vary, depending on the different geographical contexts as well as the research design. Also, due to time the limitations of the research (the first shocking experience with the pandemic situation), results should be treated cautiously. Within the evidence in Western countries, a number of studies agree on observations of certain trends of resilience concerns relating to population health, food safety and food justice [e.g., (5, 7, 26, 27)]. Regarding the effects of the pandemic on households, it is not surprising that one of the most decisive factors detected within the research which influenced consumer behavior and eating habits was income change (5, 6, 26). Especially the study by Millard et al. (5) revealed that households, fragile even before the pandemic, experienced a loss of income during Covid-19 which then made their situation worse. Other research has shown that the impacts vary across population groups with different socio-economic characteristics, but families with children are understandably identified as vulnerable groups (6, 26).

Psychological factors influencing food behavior should not be neglected. The need for stockpiling can be explained by a decrease in food shopping frequency as a result of restricted mobility and fear of infection [this trend is confirmed by Vidal-Mones et al. (28), Millard et al. (5) or Babbit et al. (29)]. In terms of the amount of food consumed, many people reported an increase in food intake, however, the problem lies in the change in diet. Consumers have experienced a decline in dietary quality–less fruit, vegetables, meat and fish were eaten, in contrast to more consumed cheap foods or those with a long shelf life–that is, industrially processed products (5–7). The need to address food availability in the future is also evidenced by the increase in the workload of food banks. From the donors’ point of view, Oncini (30) clearly reports a significant increase in the volume of donated food, confirmed by 76% of organizations in the sample. The aforementioned observation led to discussions on how to address the growing problems of obesity and diseases associated with poor eating habits such as diabetes as well as food insecurity (7, 10, 31).

## 3 Specific situation of the agri-food system and AFNs in Czechia

The agri-food system in Czechia differs from that of Western Europe, which is the consequence of decades of the communist regime in the 20th century and the post-socialist transition. The socialist model of industrial agriculture was based on destruction of private farming and an emphasis on high concentration of large-scale socialist cooperatives. After the change of the regime in 1989, economic transformation and subsequent privatization took place in Czechia (32, 33).

### 3.1 Perspective of Czech consumers

Related to retail landscapes and consumer behavior, the very dynamic processes were shaped by internationalization trends and the adoption of Western models and patterns of shopping behavior (34) leading to the enormous spread of foreign retail companies in the form of large-scale retail formats (such as hypermarkets, supermarkets, discount stores and shopping malls) and causing a radical transformation of retail networks in post-socialist cities (35). Therefore, transnational corporations have gained dominance, and partially replaced traditional smaller retail facilities (36, 37).

However, the quantitative boom was followed by a sobering of consumers caused by scandals of retail chains and information circulating in the media that supermarkets in Czechia sell food of lower quality than in neighboring countries (37). Thus, consumers choices started to be oriented not only to common and cheap products but also took into account aspects of the locality (36) and the “quality and safety” of food (38), which is a process referred to as a quality turn in the context of Western countries (39). Relatively new data on the growing interest in food quality is provided by Severová et al. (40), who state that the number of such quality-aware consumers increased from 35 to 40% within 5 years. Together with new trends of food behavior, the evolution of AFNs on the market has been underway. However, given the fact of adopting (or importing) ideas and elements of market-oriented AFNs developed in Western countries to the context of Central and Eastern European countries, researchers point out some specific local characteristics and the different evolution of AFNs due to their different settings (32, 41). Regarding AFNs coming to the Czech market during the last decades, farmers’ markets began to develop around 2010, followed by other forms of AFNs such as community-supported agriculture (CSA) or box schemes. To compare–in neighboring Germany, these forms of direct sale have been operating for more than half a century (42). However, it should be noted that some forms of markets and farmers’ shops (or collective farms’ shops during communism) have been part of the Czech AFNs for many decades, especially in rural areas.

The consumers’ side is monitored mainly via public opinion surveys. Comprehensive results are presented by the research of Spilková (36) comprising 3,168 respondents. The most popular forms were the farmers’ markets (38% of the respondents do sometimes shop there) and direct shopping at the farm gate (23% of respondents). Farm shops were used by almost 18% of respondents and box schemes were utilized by 5% of respondents. Similar results can be seen in a recent public opinion poll regarding consumers’ preference to local food (43), which showed that more than two fifths of respondents (out of a total of 1,549) at least sometimes buy local food. Among the forms of AFNs we monitored in our research too, the most popular was direct purchase from local producers (48%), farmers’ markets (47%) and local vegetable markets (44%). Pick your own food was mentioned by 31% of respondents, online option by 16% of respondents and as the latest option CSA was selected by 5% of respondents. Taking into account socio-demographic characteristics, according to Spilková (36), AFNs are in general favored by women and highly educated people in managerial positions or entrepreneurs.

Specifically, farmers’ markets experienced a big boom in the capital Prague, connected to local political support in several city quarters. Fendrychová and Jehlička (41) pointed out some disproportion between expectations, promotion on the one side and capacity of suppliers on the other side, especially at the beginning of the boom of farmers’ markets which Prague experienced in the early 2010. In less than 24 months, 41 farmers’ markets were established in and around the city, but as time went by, the numbers decreased and the market stabilized. Currently, around 140 farmers’ markets are evidenced in Czechia according to publicly available databases [e.g., (44)]. The least numerous form of sales–CSA–began to develop in Czechia in a similar period as farmers’ markets, but they are not expanding as dynamically (the same applies to box schemes). According to continual international monitoring of CSA, around 80 groups containing 5,000 people are active in Czechia, cooperating with 30 farms in 2020 (45).

Regarding other forms of AFNs, media report the increasing trend of interest in pick-your-own. Especially after the experience with the Covid-19 pandemic, initiatives were created to map the locations of farms offering this service. Furthermore, more emphasis is placed on the promotion by social networks and online sales. Unfortunately, there is no database available which would provide a comprehensive overview of AFNs activities. Partial data can be collected through different (mutually uncoordinated) initiatives that carry out surveys, however, available databases often lack updates.

### 3.2 Position of small-scale food farmers in Czechia

With regard to producers, the involvement of farms in AFNs is quite low. It may be a consequence of the large-scale agribusiness model, which remained dominant even after the post-socialist economic transition, although with private owners. Thus, the current agri-food production and consequent food processing part of the food systems is distorted due to the high concentration of big agribusiness enterprises that control the food market supply and prices. According to the statistics, nearly 30,000 farms operate in Czechia, while 60% of agricultural land is held by only 1,740 (6%) of farms which are larger than 500 ha. An overwhelming 86% of agricultural land is managed by only 5,000 (17%) of farms which are larger than 100 ha (46). Czechia is the country with the highest average area per agricultural holding: 133 ha in 2013, compared to an EU average 16 ha (47).

The main reason of such a low proportion of small and medium farms is the fact that the landowners, who received the land back in the 1990s restitutions, often sold or rented the agricultural land to a local agro-enterprise (often a previously former large socialist cooperative farm). The descendants of former farmers usually found it impossible to get back into farming after nearly 40 years of disruption. Thus, a significant number of the former socialist cooperatives at the time remained and were merely transformed into different legal forms of entrepreneurship (48). These large enterprises work better on the basis of large-scale agriculture linked to the food (and other) industry and the number of small and medium farms is relatively low which together leads to low involvement of farms in AFNs.

This could be illustrated by a case study of the Moravian-Silesian region by Hruška et al. (32). Analyzing publicly available (but not exhaustive) databases on the internet, they revealed that the proportion of farms integrated into AFNs was 1.4% of all farms in the region (5% of the total number of organic farms) in 2018. The low AFNs representation within the market is also indicated by the report of the Ministry of Agriculture, which states that the share of organic food (which is an integral part of the AFNs concept) in the total consumption of food and beverages in 2020 reached 1.8% (49).

### 3.3 Non-market aspects of AFNs based on food self-provisioning

Additionally, to market-oriented AFNs, informal food networks and non-commercial food activities are considered to be part of AFNs too by some authors. As argued by Daněk et al. (50), although AFNs is a concept dominantly developed in Western countries, many similarities can be observed with FSP, including the motivation of participants and material, social and environmental outcomes. FSP has strong roots in Europe, in the countryside as an integral part of life and in the cities especially since the 19th century as a way of ensuring food security for the growing working class (51). Informal food networks flourished during World War I as well as World War II. During the second half of the 20th century FSP was more common in countries of Central and Eastern Europe, yet it remained popular in some of the West European countries too (52) and experienced a boom in the Covid-19 pandemic (53). FSP is still a vivid phenomenon and hobby activity accompanied by a rich combination of sharing, exchanging and gifting food products (54–56). Surplus food from own production is sometimes also unofficially sold from so-called “the yard” – for example honey or eggs. These activities show a significant level of the persisting practice among the population despite the dominance of either centrally planned socialist or free-market capitalist economy.

While the concept of AFNs is associated with criticism that it appeals to consumers in cities with higher incomes and higher education (57), according to research to date, a diverse range of social groups participate in FSP. Although FSP was associated with the narrative of the so-called coping strategy, i.e., activities carried out due to the need to obtain enough food (unavailable due to lack of finance or lack of food on the market), this approach has been successfully overcome in research (50). Research of gardeners’ motivations supports this interpretative change, proving that having fresh and healthy food as well as a leisure activity are the main motivations for gardening, though financial motives cannot be fully dismissed (24, 58, 59).

The proportion of people participating in FSP in Czechia is more or less stable in time and oscillates between 40 and 45 percent of the population in the last two decades (60) with a recent increasing trend (58). Research shows that FSP is widespread among the whole Czech society, though it is more often practiced in rural areas (55), mostly due to higher availability of land for gardening in family houses with gardens. Fruit, vegetables, or potatoes are often grown but domestic animals are kept too to a lesser extent (24). Despite the focus of our study on Czechia, it should be noted that FSP is typical for other Global North countries too [e.g., (52, 61)]. Apart from the food growing itself, positive outcomes for well-being and life satisfaction are attributed to it as well (62).

## 4 Methods

The data were collected by the professional market research agency Median in October 2021. Participation in the questionnaire survey was voluntary and anonymous. A sample of 515 respondents representative of the adult Czech population was selected by quota sampling following these criteria: sex, age, education, municipality size and region. Data collection combined Computer Assisted Web Interview (online survey, CAWI) and Computer Assisted Telephone Interview (CATI) in order to include the part of the population reluctant to use the internet. Out of a total 515 respondents, 449 were CAWI and 66 CATI which reflects the share of the offline population according to the data and experience of the market research agency. See Table 1 for a complete overview of the sociodemographic characteristics of the respondents. The survey sample reflects the adult population with a very low deviation of less than 1% in most of the sociodemographic characteristics (see Table 1 in Supplementary material for a comparison of the sample and the Czech adult population according to the sampling quotas). There is only one exception in which our sample differs more from the overall Czech population–housing type. There is a higher share of households living in family houses in the sample than in reality which might potentially slightly increase the share of FSP in the results as this is commonly practiced by those with household gardens (see below). However, this characteristic was not a sampling quota. We keep this and other limitations in mind while discussing the results and summarize them in the 5.5 Limitations section.

**Table 1:** Sociodemographic characteristics.

	Respondents
Sex	
Male	48.9%
Female	51.1%
Age groups	
18–34	23.3%
35–49	32.0%
50–64	24.1%
65+	20.6%
Education	
Grammar	10.9%
Lower middle	34.6%
Upper middle	36.7%
University	17.9%
Economic activity	
Employee	52.0%
Self-entrepreneur	6.0%
Student	6.2%
Retired	27.8%
Unemployed	3.1%
Other (parental leave, homemaker, etc.)	4.9%
Household net income	
< 416 EUR	1.7%
417–833 EUR	17.5%
834–1,250 EUR	15.7%
1,251–1,666 EUR	16.7%
1,667–2,083 EUR	19.2%
2,083–3,125 EUR	15.5%
3,126+ EUR	6.1%
Do not know/do not want to say	7.5%
Children under 20 years in household (% yes)	37.5%
Municipality size	
< 1,000	16.9%
1,000–4,999	21.7%
5,000–19,999	19.8%
20,000–99,999	20.6%
100,000+	21.0%
House type	
Family house	46.2%
Apartment	51.8%
Other	1.9%

*N* = 515. Question Household net income originally had 18 categories, in this table it is summarized into 7 categories. It was recoded into quintiles (see Results and discussion for details), Municipality size was recoded to urban/rural binary variable (5,000 inhabitants as threshold) and respondents answering “Other” on the question on House type were omitted from the logistic regressions. Exchange rate 1 Euro = 24 CZK was used for the income conversion.

The questionnaire was a follow-up to the international research initiative “Our relation to food during Covid-19 pandemic”,^1^ which reflected the first wave of the pandemic in Spring 2020 in several European countries [see (5) for details]. The original set of questions focused on changes in food behavior and the perceived risk associated with Covid-19 was complemented with questions focusing on food resilience, AFNs and food growing in our 2021 survey. It reflects food behavior after the experience of three pandemic waves with peaks in October 2020, January, and March 2021 and before the start of the fourth wave peaking at the end of November 2021. Since the start of the pandemic in March 2020, the Czech government declared a state of emergency several times, associated with specific anti-epidemic measures impacting the food sector and households (including the repeated ban on farmers’ markets, strict hygiene regulations, mandatory vaccinations for entering restaurants, etc.).^2^ Given the timing of our survey, it detects the main trends in light of adopting anti-pandemic measures and changing socio-economic conditions in Czechia, mainly starting with the increase of food prizes.

Given the main themes of the research initiative mentioned above, the whole questionnaire consisted of sections focusing on the following thematic areas:

- food shopping (particular types of shops and direct purchase from producers), changes in shopping frequency during the pandemic,
- food behavior (consumption of specific types of food, changes during the pandemic, reduced consumption for financial reasons, food anxiety, food donations),
- food self-provisioning (access to a garden, attitude to food self-provisioning, assessment of the importance of food self-provisioning in total consumption).

This paper presents results of the analyses of answers to the selected topics: types of shops used with a special focus on the types of AFNs, changes in food-related behavior (food security, food consumption, food shopping) and food growing. The particular wording of all analyzed questions as well as frequencies of the answers in per cent can be found in Supplementary material.

Statistical analysis of the data was processed with IBM SPSS 28 software. Methods include descriptive statistics, Pearson correlation, crosstabulations with Chi-square and logistic regression. If not stated otherwise, the statistical significance mentioned in the text is on the level of 5%.

## 5 Results and discussion

The following sections respond to the research questions and the results are discussed with the findings of other foreign and domestic research as well as public opinion polls. Section 5.1. and 5.2 respond to the first research question: *To what extent do consumers use alternative food networks to acquire food?* Section 5.3 analyses in more depth the participation of households in AFNs with respect to sociodemographic indicators. Additionally, an analysis is conducted on the spatial perspective that differentiates households in rural and urban areas. Section 5.4 focuses on answering the second research question: *How have food acquisition and eating habits changed in the context of the pandemic?*

### 5.1 Food purchase

In this section, the situation in the food market is examined in more detail: where people buy food and how many of them use market-oriented AFNs. Two separate questions focused on fresh fruit and vegetables and other fresh food (dairy and bakery products, meat, pastries, etc.) are combined in Table 2.

**Table 2:** Where food is being purchased.

Type of shop	YES, any fresh food	Yes, detailed			No
		Both	Fruit and vegetables only	Other fresh food only	
Super/hypermarkets	91.5%	80%	6.8%	4.7%	8.5%
Small shops	56.9%	30.5%	5.8%	20.6%	43.1%
Cooperative shops	6.4%	2.1%	0.2%	4.1%	93.6%
Organic/local/healthy food shops	8%	3.5%	2.9%	1.6%	92%
Farmers’ and other markets	21.6%	8.3%	10.5%	2.7%	78.4%
Local growers and producers	19.6%	6.8%	9.7%	3.1%	80.4%
Home delivery (online or phone order)	7.8%	5%	1.7%	1%	92.2%
Other	7.0%	1.6%	4.9%	0.6%	93%

Each type of shop was questioned separately (yes/no) sum of these two answers is 100%. *N* = 515.

The results show that supermarkets and hypermarkets are the most common place for food shopping, confirmed by almost 92% of respondents. It is likely that the process of consolidating the dominant position of large retail chains, lasting several decades, is continuing.

According to Ratinger et al. (38), in 1997 a quarter of Czech households shopped in large retail chains and by 2013 it was 86%. Therefore, it seems realistic that this number continued to grow. Small, specialized shops such as greengrocers, butchers or bakeries still maintain their position with over half of the population visiting them. Three of the eight types of shops were classified as AFNs: organic/local/healthy food shops, markets (including traditional markets where food is sold by resellers and the origin of the food is not addressed as well as new farmers’ markets) and direct purchase from producers.^3^ As Table 2 shows, markets and direct purchase from producers are more popular than specialized shops with organic, local and healthy food. When summarized, the share of respondents who buy either fruit and vegetables or fresh food (or both) in any of the three types of alternative food network shopping places is 37.5%. This group is hereafter referred to as AFNs shoppers.

When focusing on the direct purchase from producers only, (19.6% of the sample, see Table 2), the vast majority of this group (72%) buy food directly at the farm gate sale or in the farm shop. Farm gate sale seems to be organizationally simple and financially advantageous for both clients and sellers, moreover, the opportunity to visit farmers and see with their own eyes where their food comes from is very attractive for customers. This may be related to consumers’ need to obtain food from a trusted source, because as the study by Carfora and Catellani (63) states, trust is one of the three decisive factors (along with availability and health) motivating consumers to buy local food.

Regarding direct sales, it is also necessary to mention the so-called hybridization of AFNs, which in the post-socialist environment manifests itself in the involvement of large farms in direct sales to consumers [described in the aforementioned study by Hruška et al. (32) or Fendrychová and Jehlička (41)]. Here it is necessary to say that farm shops and farm gate sales were already used in state enterprises during socialism, so consumers are used to it. However, there is a certain dilemma, how to identify this model from the point of view of AFNs–there is a rapprochement between the consumer and the producer, but the way of managing the business can be entirely conventional. In our research, direct purchase at farm gate or in farm shops is considered to be participation in an AFNs, regardless of the size or type of farm. We find the direct contact between producers and consumer most important from the perspective of this study.

Other popular ways of purchase are based on online shopping with a different way of delivery: via social networks and flexible delivery (17%), online order with pick up at the farm (12%), regular box scheme with delivery to specific place of distribution (11%) and regular box scheme with delivery to home (10%). We assume that online marketing will become more important, and it could be a suitable strategy for farms in the future to maintain their market position and reach new customers, which is already observed in the aforementioned research [see (64, 65)]. Another form of purchase was self-picking on the field (10%) which has become heavily promoted in advertisements in the last 3 years and is most often used when selling strawberries and apples. Also, 9% of respondents referred to the open answer “other forms” mostly without clarification. Finally, minimal interest was found in relation to CSA (1%),^4^ which corresponds to the above-mentioned literature demonstrating the lagging behind of CSA compared to other forms of AFNs (43, 45). It seems like Czech consumers have not become accustomed to closer and more binding cooperation with the producer to a large extent. Despite low distribution, the local and solidarity-based partnerships show a high level of allegiance even in times of crisis. CSA and similar models reported high resilience: 90% of them confirmed in a global survey (among 40 countries including Czechia) that they did not experience any interruption of deliveries at all. On the contrary, they experienced a slight increase in the interest of newcomers in joining them (66).

Considering the situation of the local food market from the point of view of consumers, our results and other studies indicate that there is a group of consumers interested in local food, but the local food market is not sufficiently developed. Moreover, farmers or producers oriented to direct sales find it difficult to compete with commodities from the conventional market controlled by large business entities. The problem with the local food market and barriers hindering its development is illustrated by the opinion of the Czech public reported in a survey by Hanzlová (43), in which 61% of respondents perceive a lack of local producers and farmers in the vicinity of their residence. Furthermore, 91% of the respondents think that support for local food is insufficient (at the level of the EU, Czechia, regions and microregions). In addition, they perceive cheap food from foreign sources and large agricultural enterprises as the main problem obstructing the production and sale of local food.

### 5.2 Food self-provisioning

**Figure 1:**
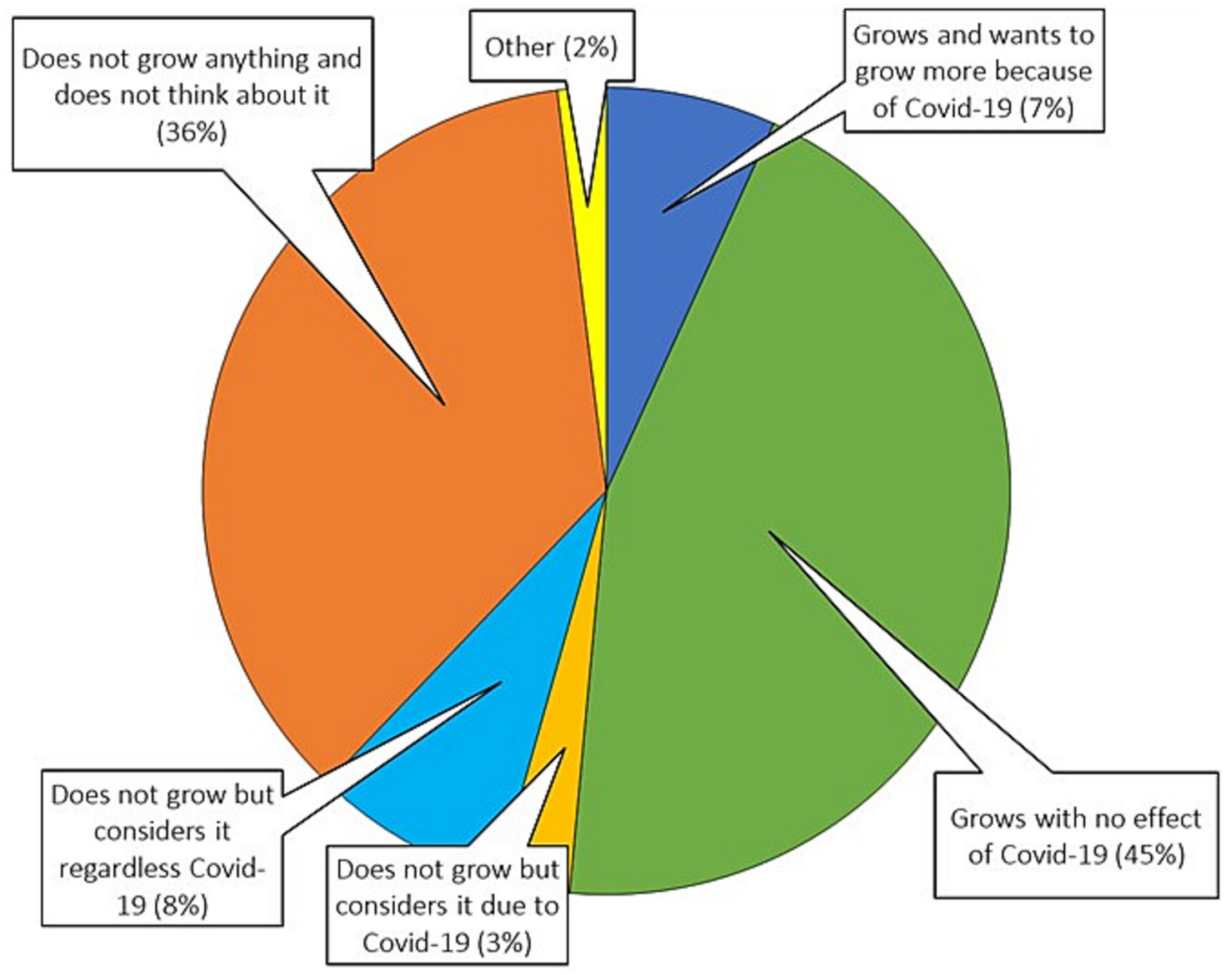
Households’ approach to FSP. Sum may not be 100% due to rounding. *N* = 515.

Another area of interest are AFNs operating outside the market environment, i.e., in our case food self-provisioning. As mentioned above, FSP can include keeping domestic animals. In this text we often use the term “grow food” for the sake of simplicity which can include keeping animals too (see Supplementary material for exact wording of the questions). A major FSP-related question asked respondents to choose the answer best matching the relation of their household to own food production (see Figure 1). The overall share of respondents who grow fruit and vegetables (or keep domestic animals for food) is 51.5%. This number is accompanied by another 11.7% of respondents who consider growing food in future. While some of the respondents who chose option “Other” also mentioned occasional food growing, we consider them as non-growing to be rather conservative and respect their perspective.^5^ Taking into account the results in other studies, the increasing interest in growing own food is confirmed also by Millard et al. (5), who recorded this trend in both rural and urban areas. As for studies beyond the European context, in Canada, Mullins et al. (67) records an increase in new people interested in their own food production, especially in urban areas. Another study describes a growing interest in food self-provisioning in New York, with respondents’ clear intention to strengthen control over food availability (68). Further, Egerer et al. (69) dealt with FSP during the pandemic and emphasized the importance of this activity in relation to financial savings. In addition, they revealed a link between the growing interest in gardening and concerns about in-store shopping and access to fresh food.

To evaluate the importance of FSP in terms of amount of produced food and to assess the level of food resilience, respondents were asked to indicate how important is their own production compared to food shopping (in terms of fruits, vegetables, potatoes, eggs, etc.) (Table 3). Growing fruit and vegetables is a supplementary source for 42.6% of food growers and for another 40.8%, it is only a negligible seasonal source. For nearly one-fifth (17%) of food growing households, it is a major source of food comparable to shopping (answers majority + essential). We are aware of the fact that it is a subjective estimate of the respondents, yet it is supported by previous research, both in local case studies as well as in large surveys using different assessment methods, including questionnaires, interviews or food logs (23, 24, 70). FSP can be seen as a significant source of fresh and healthy food. When recalculating the households for whom FSP is a major or essential source of food as a share of total population, it is 8.8% of households. Such a number is slightly higher than the share of households shopping via home delivery or visiting specialized shops with organic, local and healthy food.

**Table 3:** The importance of own food in total food consumption.

Respondent’s answer	%
Covers majority of food consumption	6.8%
Essential source, comparable with shopping	9.8%
Supplementary source	42.6%
Negligible seasonal source	40.8%
No importance	0.4%

*N* = 265 (food growing households only).

Respondents were also asked whether their households have access to a place where fruit or vegetables can be grown. Various possibilities were offered in the questionnaire (answers yes/no). Most of the respondents who grow food have access to a private garden (69.8%). The following accessible places where food could be grown include weekend house garden (18.9%), balcony or indoor gardening (17%), gardens of relatives (13.6%) and allotment gardens (7.5%). Community gardens are rather rare (0.8%). Similarly as in the case of food shopping, respondents often have access to more than one type of garden, and therefore the sum in per cent exceeds 100%. This confirms previous results showing that home gardens are the most important place for food growing (58), yet they tend to be less researched than more politicized gardens often being in public spaces, like allotments and community gardens.

Considering the evidence of growing interest in FSP, a question of availability of land for growing, especially in cities, emerges. The preservation and development of allotment and community gardens enabling food growing to those inhabitants who do not have access to their own garden should be a part of food policies. Municipalities ought to allow long term lease contracts of public land to enable the cultivation of fresh food and enhance local food production (13, 71, 72). In this regard, there is a significant gap in the strategies of Czech municipalities. Although the Gardening Act No. 221/2021 Coll. approved in Czechia in 2021 recognizes gardening as a publicly beneficial activity, there are many misconceptions in the practice of creating strategic documents. As stated by Pixová and Plank (73), who focused on the research of relevant documents in the Czech metropolises Prague and Brno, city leaders tend to understand the environmental, recreational and aesthetic benefits of gardens. However, they neglect the real function of food production for consumption which can be significant for some households and is relatively high in general (23). While community gardens are coming to the fore as a trend from the Western countries, they are still a minor phenomenon in Czechia, as our research shows. They are often seen as something trendy even by local politicians and planners who paradoxically oppose traditional forms of FSP, like allotment gardens, which are well established and productive (70). Often, allotment gardens in the cities are facing extinction to make way for development projects. An epistemic rehabilitation^6^ of traditional FSP types, like allotment gardens, in the minds of politicians and planners is needed to enable a more critical attitude toward many housing and commercial real estate projects. So far, the arguments to keep the allotments and other places for potential food growing undeveloped are raised mostly by local residents, academics or NGOs which is not sufficient.

### 5.3 Households participating in AFNs

Our findings show that AFNs shopping is practiced by 37.5%, while food growing by 51.5% households. Both of these can be obviously done by the same households. Table 4 shows how these activities are related to each other. Two thirds of households participate in any kind of AFNs, be it market or non-market oriented. Pearson correlation of binary variables AFNs shopping and food growing does not reveal any significant relationship but a slight positive trend suggesting that participation in one activity is linked to the other (*r* = 0.086, *p* = 0.52). Based on this finding, we can argue that AFNs shopping and food growing are not mutually exclusive (i.e., participating in one activity would limit participating in the other) and tends to be mutually supporting, though we have to be careful with this conclusion. This is a different finding than, for example, results of a US study by Schupp and Sharp (61) who found a significant positive relationship between participation in local food systems shopping and FSP.

**Table 4:** AFNs shopping and food growing in per cent.

		AFNs shopping	
		Yes	No
Food growing	Yes	21.4%	30.1%
	No	16.1%	32.4%

*N* = 515.

The effect of sociodemographic characteristics was tested by logistic regressions with binary dependent categories AFNs shopping and food growing and independent sociodemographic as binary or categorical variables. Some characteristics were used in the form shown in the Tab. 1 (sex, age group, education, children under 20 in the household). Income was transformed into quintiles for the purpose of the analysis. This was done by dividing the mean of the income category in the questionnaire by the weighted number of household members^7^ and then transforming into quintiles. Narrowly understood economic activity was limited to binary categories of active (employee, self-employed) and inactive (student, retired, unemployed, other). Similarly, place of living was also reduced to binary category rural/urban with municipality size of 5,000 inhabitants as the distinction between rural and urban.^8^ In the case of house type, category “Other” with a small number of cases was omitted from the logistic regression.

Regarding AFNs shopping, place of living was the only one significant independent variable (*β* = 1.809, *p* = 0.011) showing higher probability of AFNs shopping in urban areas. However, the overall explanatory power of the regression was weak (Nagelkerke *R*^2^ = 0.040, *N* = 467 due to some missing values). The regression explained more variability in the case of food growing (Nagelkerke *R*^2^ = 0.315, *N* = 467) with two independent variables being significant: house type (*β* = 0.153, *p* < 0.001)^9^ and place of living (*β* = 0.559, *p* = 0.018) showing that households living in family houses as well as in rural areas have greater chance to grow food.

**Figure 2:**
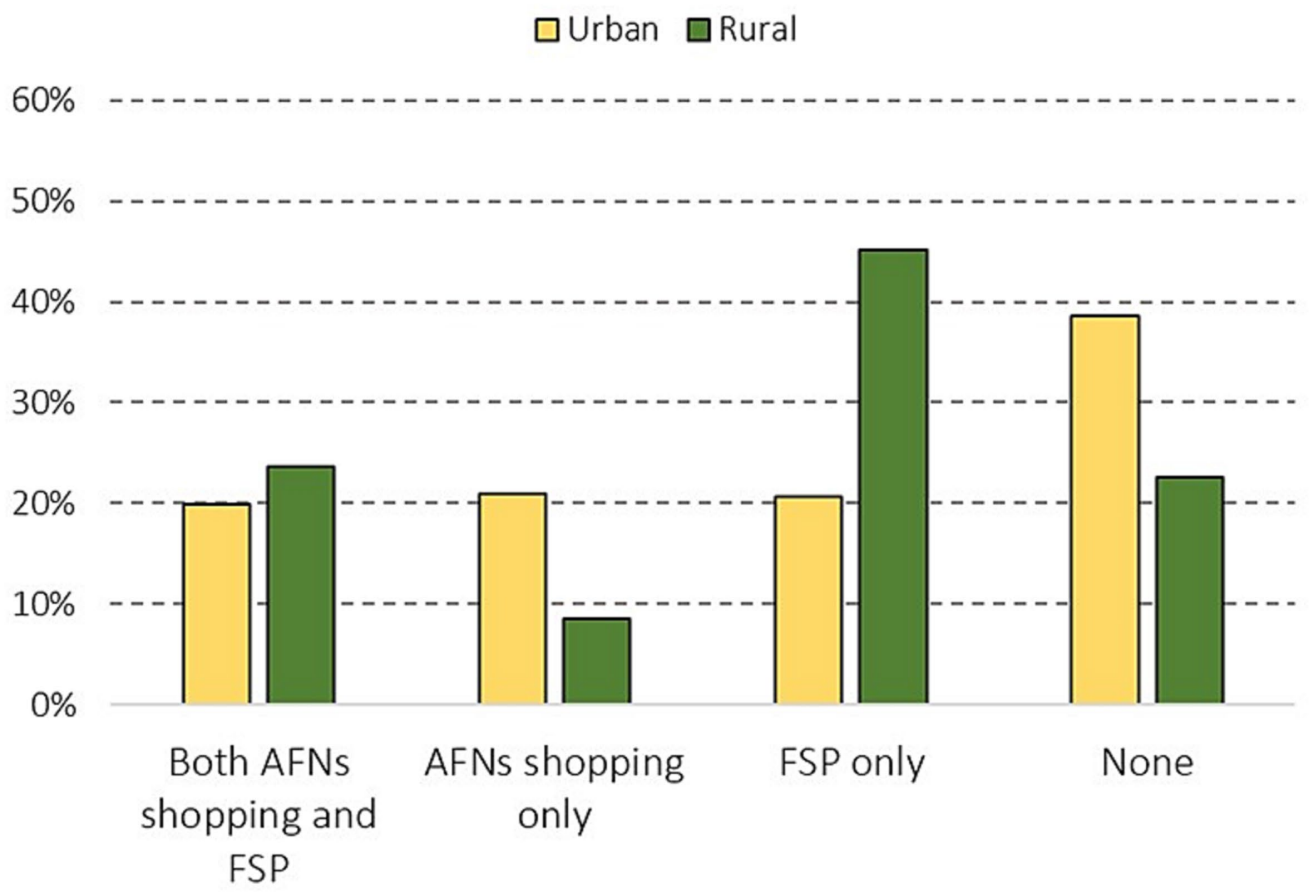
AFNs shopping and food growing in urban and rural areas. Overall significance tested by Chi-square (*χ*^2^ = 46.616, d.f. = 3, *p* < 0.001). Differences between urban and rural areas are statistically significant for all categories (Adj. Res. ≥ 1.96; *p* < 0.01) except participating in both AFNs shopping and FSP.

When assessing the weak effect of sociodemographic factors, an issue of discrepancy between individual characteristics and questions aimed at behavior of the whole household may arise. This aspect may have some influence, yet the households tend to be socially coherent units with similar characteristics of its members. In the case of FSP, previous research supports our findings about the low effect of sociodemographic [e.g., (55, 59)]. Thus, based on our results, we argue that both AFNs shopping and food growing are socially inclusive practices widespread among various social groups. Given the fact that place of living is the only variable affecting both AFNs shopping and food growing, we show the comparison of them for urban and rural areas separately in Figure 2. The group of households participating in both market AFNs and FSP is similar in urban as well as rural areas, while shopping at AFNs only or no participation in AFNs is more common in urban areas. On the contrary, sole food growing is much more frequent in rural areas.

The different distribution of AFNs shopping and FSP in urban and rural areas might have consequences for farmers. As Hruška et al. (32) argue, widespread food growing in rural areas may be seen as a competition for fruit and vegetable-oriented farmers because people living in rural areas are used to growing their own food. Our research supports this statement by showing a large group of food growers who do not shop in AFNs, yet it does not mean that the food growing households participate less in AFNs shopping in rural areas (*r* = 0.068; *p* = 0.338). The link between food growing and AFNs shopping is stronger in towns and cities than in rural areas as the correlation shows significant results (*r* = 0.141; *p* = 0.012). Despite the share of households not participating in any of the two types of food gathering is higher in urban areas (38.9 to 22.6%), the overall number of households being involved in AFNs shopping is higher (40.8 to 32.1%). Given the generally high level of urbanization of the country, developing AFNs in towns and cities, or attracting urban dwellers to direct purchase at the farm gate, seem to be a vital business strategy for farmers.

The foregoing findings addressing the potential barriers to the development of the local food market are reinforced by findings from a study by Carfora and Catellani (63), who identified availability as the most important element in local food decision-making. This is valid both in terms of seasonality and of space, as the possibilities differ in urban and rural settings. While consumers in the countryside are closer to individual farms, the offer in the city is generally more varied, as products concentrate at distinct sales points. They are, however, generally more expensive.

### 5.4 Changes during Covid-19

In the following section three aspects of changes during the Covid-19 pandemic are analyzed: financial aspects, changes in food shopping and food consumption. In each case, the effect of participation in AFNs shopping as well as FSP is analyzed. This allows us to answer the second and third research questions (changes in food-related habits and its policy implications). Full descriptive statistics of the categories of answers are shown in Tables 5, 6, including the significance of Chi-square analysis of the relationship between changes during Covid-19 and the separately tested effect of participation in AFNs shopping and FSP. Changes during Covid-19 were originally measured on Likert-like scales with 3- or 5-categories and were recoded to binary or 3-categories variables for the analysis (see Supplementary material).

**Table 5:** Income change, food anxiety and shortages, food donations.

Income change					
	*Decrease*	*No change*	*Increase*	Effect of AFNs shopping	Effect of FSP
Income during Covid-19	28.9%	59.4%	11.7%	*p* = 0.586	*p* = 0.286
*N* = 515.					

Food anxiety and shortages					
	*Often*	*Sometimes*	*Never*	Effect of AFNs shopping	Effect of FSP
Food anxiety	4.9%	30.9%	64.3%	*p* = 0.474	*p* = 0.121
Food shortages	2.7%	15%	82.3%	*p* = 0.990	***p* = 0.047**
*N* = 515. Respondents from food growing households report more often that they have never experienced a lack of food (Adj. Res. = 2.3; *χ*^2^ = 6.126; d.f. = 2). Bold text highlights *p* < 0.05.					

Food donations					
	*Important source*	*supplementary/negligible source*	*No use*	Effect of AFNs shopping	Effect of FSP
Food banks	1.6%	3.1%	95.3%	*p* = 0.289	*p* = 0.317
Charities	0.8%	3.1%	96.1%	*p* = 0.098	*p* = 0.293
Restaurants/Apps	1.4%	7.2%	91.5%	*p* = 0.886	*p* = 0.266
Relatives	3.5%	31.7%	64.9%	*p* = 0.572	*p* = 0.112
Friends	0.8%	20.8%	78.4%	***p* = 0.046**	*p* = 0.088

*N* = 515. Original five categories were recoded into three: Important includes covering most consumption and essential source; middle category merging supplementary and negligible source and no use remaining the same. Only one case shows statistical significance (*χ*^2^ = 6.148; d.f. = 2), AFN shoppers reporting food donations from friends being supplemental/negligible source more often (Adj. Res. = 2.4) and no use less often (Adj. Res. = −2.3). Bold text highlights *p* < 0.05.

**Table 6:** Changes in food shopping and food behavior.

Changes in food shopping					
	*Decrease*	*No change*	*Increase*	Effect of AFNs shopping	Effect of FSP
Supermarkets	22.7%	62.1%	6.6%	*p* = 0.302	*p* = 0.895
Small shops	8.7%	35.9%	12.2%	***p* = 0.005**	***p* < 0.001**
Cooperatives	0.8%	3.9%	1.7%	*p* = 0.797	*p* = 0.696
Organic	1.9%	4.5%	1.6%	–	*p* = 0.850
Markets	6.2%	10.9%	4.5%	–	*p* = 0.536
Local producers	2.7%	11.7%	5.2%	–	*p* = 0.131
Online	0.8%	2.9%	4.1%	*p* = 0.953	*p* = 0.074
N ranges from 33 (Cooperatives) to 471 (Supermarkets) due to the fact that only respondents shopping in a particular type of shop answered this question. However, the percentage relates to all respondents (*N* = 515) to reflect how many households do not use the particular type of shop (see also Supplementary material). The results are calculated as mean of original 5-item scale of fresh fruit and vegetables and other fresh food which was then recoded into three categories. There are positive effects of both AFN shopping (*χ*^2^ = 10.411; d.f. = 2) as well as FSP (*χ*^2^ = 16.752; d.f. = 2) on using small shops. In both cases, households participating in AFN shopping or FSP more often reported an increase of shopping frequency (Adj. Res. = 2.1 in both cases) and less often reported its decrease (Adj. Res. = −2.8 and −3.9). Bold text highlights *p* < 0.05.					

Changes compared to before Covid-19					
	*Decrease*	*No change*	*Increase*	Effect of AFNs shopping	Effect of FSP
Amount of food consumed	17.1%	72.6%	10.3%	*p* = 0.967	*p* = 0.465
Amount of food from local growers and producers	16.1%	73.4%	10.5%	***p* < 0.001**	***p* = 0.003**
Amount of organic food	21.9%	69.5%	8.5%	*p* = 0.098	*p* = 0.653
*N* = 515. Original five categories were recoded into three. There are positive effects of both AFN shopping (*χ*^2^ = 19.374; d.f. = 2) as well as FSP (*χ*^2^ = 11.336; d.f. = 2) on the amount of food from local producers and growers. Note: Respondents participating in AFN shopping increased the amount of food from local producers more often (Adj. Res. = 4.4) and less often reported no change of the amount (Adj. Res. = 2.2). This is expectable, yet we have included all respondents into the analysis, as AFN non-shoppers may buy some local food in other types of shops. Food growing respondents less often reported a decreased amount of food from local producers (Adj. Res. = −3.3). Bold text highlights *p* < 0.05.					

Food consumption decrease					
	*Yes*	*No*		Effect of AFNs shopping	Effect of FSP
Fresh fruit and veg	9.5%	90.5%		*p* = 1.000	***p* = 0.016**
Fresh meat	10.1%	89.9%		*p* = 0.763	***p* = 0.028**
Fresh fish	16.5%	83.5%		*p* = 0.714	***p* = 0.043**
Bread and bakery	2.7%	97.3%		*p* = 0.781	*p* = 0.105
Milk and milk products	4.5%	95.5%		*p* = 0.830	*p* = 0.053
Note: *N* = 515. Regardless of how often they consume the particular type of food. There is a positive effect of FSP on consumption of several types of food: food growing households report a decrease of consumption of fresh fruit and vegetables (Adj. Res. = −2.5; *χ*^2^ = 6.091; d.f. = 1), fresh meat (Adj. Res. = −2.3; *χ*^2^ = 5.153; d.f. = 1), fresh fish (Adj. Res. = −2.1; *χ*^2^ = 4.307; d.f. = 1) less often. The same non-significant trend applies to milk consumption. Bold text highlights *p* < 0.05.					

Frequency of food consumption					
	*Less than once a week*	*1–3 days a week*	*4 days a week or more*	Effect of AFNs shopping	Effect of FSP
Fresh fruit and veg	4.5%	33.4%	62.1%	*p* = 0.164	***p* = 0.008**

Note: *N* = 515. Regardless of how often they consume fresh fruit and vegetables. There is a positive effect of FSP on the frequency of consumption of fresh fruit and vegetables (*χ*^2^ = 9.540; d.f. = 2). Respondents from food growing households eat this type of food more often 4 days a week or more (Adj. Res. = 3.0) and less often 1–3 days a week (Adj. Res. = −2.3). Bold text highlights *p* < 0.05.

#### 5.4.1 Financial aspects of Covid-19 and its impact on food security

Within our sample, 28.9% of respondents confirmed a decrease in household income during Covid-19, while only 11.7% reported an increase (see Table 5). Contrary to this, 13.8% confirmed a reduced amount of money spent on food but increased expenses were confirmed by 37.7% of respondents. To clarify the situation in terms of food security, we asked for concerns about food shortages, which were confirmed by more than a third of respondents (4.9% often and 30.9% sometimes). The real experience with a lack of food applied to a smaller, yet relatively high, share of respondents: 2.7% reported a lack of food in the household often and another 15% sometimes. We were also interested in the group of respondents who confirmed the use of food donations–the use of resources such as food banks, charities or restaurants/apps was confirmed by 3.9–8.6% of respondents, of whom only a minority stated that it was an important source of food and the rest of them identified these sources as a supplementary/negligible source. Food donations from relatives or friends was more common with 35.1%, respectively 21.6% of respondents receiving some food. Similarly as in the case of food from institutions, it was only a supplementary or negligible source of food for the vast majority of the respondents.

It follows on from previous research showing that food is often donated by friends or relatives who grow the food themselves (55, 58). There were only two cases that showed a significant effect of AFNs shopping or FSP. First, food growing households experienced food shortages less often than non-growing households. Second, AFNs shopping households used food donations from friends more often as supplementary/negligible source of food than other households. The significance was right below the 5% threshold, yet it suggests some positive effect from being involved in AFNs.

#### 5.4.2 Changes in food shopping and consumption

The time of the pandemic brought new influences on consumer behavior; therefore, respondents were asked to evaluate changes (decrease or increase) in the frequency of visits to selected shopping places compared to the time before the pandemic. The results do not indicate significant changes and these results might indicate the good accessibility of conventional shops in general (as Table 6 shows). However, Lichter and Malý (35) found that government restrictions had different impacts on (not only) food purchase accessibility in cities, depending on the various types of urban structure that implies diverse forms and assortment of retail units. Thus, the authors argue that more comprehensive retail planning that reflects the potential local impacts of global crises is essential to ensure greater spatial equity in access to basic daily commodities. Regarding AFNs, a change was noted for farmers’ markets, where 6.2% of respondents indicated a decrease in the frequency of visits while 4.5% reported increase. It is difficult to assess the causes and the validity, because in Czechia farmers’ markets were banned in certain phases of the lockdown in 2020 and 2021 as part of anti-pandemic measures. With regard to the wider European context (including data from Czechia), other signs of the unfavorable situation of AFNs are reported by Millard et al. (5), showing a significant decline in purchases in AFNs shops (e.g., farmers’ markets, street markets, cooperatively owned or solidarity shops, specialist organic food outlets and buying food directly from the producers) during the pandemic. Although, the authors admit this may be affected by the period when data were collected too because the harvest period had not yet fully begun (Spring 2020). The only one type of shop in our 2021 research whose use was affected by AFNs shopping or FSP were small shops. Households which visit AFNs shops as well as those which grow food reported increased frequency of visiting small shops.

Regarding changes in food consumption, 17.1% of respondents reduced their overall food intake, which may not be the result of financial problems, but if we take into account that 21.9% of respondents confirmed a reduction in the amount of food of organic quality, we can conclude that a certain group tried to reduce expenses in the area of food. Financial uncertainty on the part of consumers leads to a reduction in the purchase of organic food, which is traditionally more expensive than those from conventional production (8). Therefore, the observed reduction in the consumption of products of organic origin can be considered a threat in terms of the development of the diet’s quality and the sustainability of the agri-food system. The reduced interest in organic food is confirmed, for example, by Millard et al. (5), where a decrease of up to 10% is recorded, especially in households with income loss. However, this trend, in particular, will vary between social groups, as suggested by a study by Profeta et al. (6), who state that changes in both directions–decrease and increase–are mutually compensating, so there is no radical change.

In addition, 16.1% of all respondents reported a reduced amount of food produced by local producers or growers. Shopping in AFNs as well as participation in FSP had some effect. Those shopping in AFNs have increased amount of local products purchased more often and food growers reported at least a limited decrease. Thus, consumers involved in at least some form of AFNs (even if it is a form outside the market) seem to be more loyal to the local products [*cf.* (77)].

With respect to the consumption of specific types of food, consumption was reduced in case of fresh fish (16.5% of respondents), fresh meat (10.1%) and fresh fruit and vegetables (9.5%), while consumption of milk and dairy products (4.5%) as well as bread and bakery (2.7%) were barely affected. Consumption of fresh fruits and vegetables appears to be a striking issue with regard to health and nutrition. Among our respondents, only 35.1% confirmed that they consume these foods daily, while 27% do 4–6 times a week, 23.9% only 2–3 times a week and the remaining 14% once a week or less. At the same time, the World Health Organization recommends the consumption of 400 g of fresh fruit and vegetables per day. The media also draw attention to this problem in Czechia [see, e.g., (46, 78)]. Therefore, more attention should be turned to ensuring food and nutrition security, with a focus on vulnerable groups of inhabitants, such as low-income families, children, elderly, etc. (4, 16). It is worth noting that food growing is associated with a higher frequency of consumption of fresh fruit and vegetables as well as a less frequent drop in the consumption of fresh meat, fresh fish and already mentioned fresh fruit and vegetables. Our results cannot prove causality, however, they suggest that there is a link between FSP and healthy food as well as FSP and stability of diet though it can be indirect.

### 5.5 Limitation

Nevertheless, our study has several limitations: despite reflecting a representative proportion of the Czech population, the sample size of 515 respondents was not so robust. Thus, the results need to be treated with caution when generalizing. At the same time, we allow for possible distortion due to the prevailing online survey compared to the telephone one. Generalisability of the effect of sociodemographics is limited due to the fact that individual respondents were surveyed but both shopping and food growing are often activities of more household members. Furthermore, the study is limited in time and it would be advisable to repeat it for a more accurate identification of trends. The exact share of food growing households might be slightly affected by the overrepresentation of respondents living in family houses in our sample (yet this could at least be partly balanced by not considering “Other” respondents from Figure 1 as food growers). Finally, the questionnaire asked for frequency of shopping or fruit and vegetables eating but not for the exact amount.

## 6 Conclusion

Based on our main findings, large retail is the dominant source of fresh food for Czech households. Supermarkets, hypermarkets and discounts are visited by the vast majority of the population. AFNs are also widespread with two-thirds of respondents’ households being involved in some form of them, be it market or non-commercial activity (shopping and food growing). These two activities tend to reinforce each other and show a high level of resilience with only small deviations in times of crisis. The results suggest that households have various ways how to get fresh food which is a positive aspect supporting food resilience. When differentiating between market and non-market AFNs, food growing is more widespread than shopping (51.5% compared to 37.5% of respondents) which reflects the traditionally strong position of FSP as well as specifics of the agri-food system. Given the similar findings of previous research, we conclude that market-oriented AFNs are still underdeveloped in Czechia with cheap food imports and the strong position of large agricultural entities being a barrier to its development. The findings related to FSP, including food sharing, the amount of self-produced food and another 11% of respondents who consider growing in future, make it an important activity which should not be neglected in the sustainability and food policies.

The effect of the Covid-19 pandemic was manifested mainly by the financial problems and to a lesser extent (but visible) by the decrease in food consumption, quality food orientation and AFNs’ participation. This shows that in times of trouble, the need to cut expenditures can indirectly affect farmers and food producers selling through AFNs. A striking finding is the low frequency of fresh fruit and vegetables consumption. Despite the drop due to Covid-19 being evident but not crucial, general consumption was quite low before the pandemic and could be expected to lower even more because of rising food prices since October 2021 when the data were collected.

The above-mentioned aspects relate to the resilience of households and food systems as well as nutritional and therefore also health issues. This paper contributes to the current debates with a case study of a Central and Eastern European country, particularly with the novel approach linking together market and non-market AFNs, additionally putting them into the context of the pandemic and potentially also post-pandemic changes.

Based on our study, we suggest several implication and summarizing recommendation for a complex food policy: 1/ An urgent need for policy support of local agri-food value chains is needed, especially via empowerment of small and medium farms to strengthen the abilities of actors of local or regional agri-food supply chains to respond more appropriately to adverse events and to keep these locally oriented systems more resilient. Inspiration is offered at the global level. FAO (13) recognizes the importance of local food systems for ensuring local food accessibility and perceives local food production as a key measure to mitigate the negative impacts of crises. FAO recommends promoting local food production and short supply chains and a higher degree of local or regional self-sufficiency. The argument for integrated food policies with a wider range of stakeholders participating in their design and evaluation and with more balanced power relations is supported by De Schutter (79) as well. It requires a transition from understanding food as a commodity to a common good, including the roles of farmers as stewards of the agricultural landscape and a close community (79, 80). At the state level, logistics and distribution issues should be addressed to encourage the availability of local food (63).

On a regional scale, we point out that a great challenge for local or regional food policies lies in a systematic development of urban and peri-urban agriculture that contributes to regionally based resilience of agri-food territorial systems. Municipalities and regional authorities need to find feasible ways how to preserve agricultural land around cities via spatial planning and various sets of policy instruments, including support of public procurement to enable local food production and consumption (especially fresh fruit and vegetables) based on short distances and tight bonds between producers and urban consumers (5, 81, 82).

2/ Food policies to support sustainable food consumption should widen the perspective of citizens as consumers as well as producers. Findings from our study show the insufficient consumption of fresh fruits and vegetables on a daily basis to be alarming. According to our results, the consumption of these foods has fallen even further during the pandemic, and considering the extremely quickly rising prices since the questionnaire survey, there is no prospect of improvement. Therefore, more attention should be paid to ensuring food and nutrition security, with a focus on vulnerable groups of inhabitants, such as low income families, children, elderly, etc. (4, 16, 63). It is necessary to make sustainable and healthy diets more affordable and accessible via a mix of policy measures, including educational programs and the presentation of good practices to improve food literacy (83). Furthermore, policymakers can increase consumer trust in local foods by raising the understanding of the health advantages and environmental sustainability.

Our results confirmed that food self-provisioning is an important part of the food system as a source of quality food. Especially in urban areas, the preservation and development of allotment and community gardens enabling food growing for those inhabitants who do not have access to their own garden should be part of food policies ensuring quality food and nutrition security. Municipalities ought to allow long term lease contracts of public land to enable the cultivation of fresh food and enhance local food production (13, 71, 72). In Czechia, support of gardening by municipalities and the Ministry of Agriculture is explicitly designated in Gardening Act No. 221/2021 Coll. and should serve as a practical tool.

Considering upcoming research agenda, our findings could be addressed by future work which can build on this paper and previous studies of AFNs and FSP in various regions of the world. Particularly, the households’ response to the increasing food prices and the role of AFNs and FSP and combining of food from various sources should be studied to understand food resilience on the household level. On the higher level of regional food systems, a specific focus on different forms of AFNs and their popularity should be taken to account too, similarly like in the case of FSP, identify those more popular and take the long-term experience or local conditions into account by both sellers and authorities setting the policy frameworks.

## Data availability statement

The raw data supporting the conclusions of this article will be made available by the authors, without undue reservation.

## Author contributions

ZS: Conceptualization, Formal analysis, Investigation, Methodology, Supervision, Writing – original draft, Writing – review & editing. JV: Data curation, Formal analysis, Investigation, Methodology, Validation, Visualization, Writing – review & editing. BD: Funding acquisition, Investigation, Methodology, Writing – original draft, Writing – review & editing.

## Footnotes

^1^https://www.food-covid-19.org/

^2^The first state of emergency (12 March to 17 May 2020) was accompanied with very strict measures. Only shops with basic assortment, including food, were opened and canteens remained open to serve takeaway. The second state of emergency (5 October 2020 to 11 April 2021) applied similar measures, but not as strict. Restaurants, cafes and canteens had limited opening hours (except for children, vaccination certificate needed) and shops remained more or less open. In the discussion section we use some of these events to contextualize the results of our survey.

^3^It is worth noting that the “cooperatives” are not considered to be part of AFNs in this study despite the fact that there is a network of stores based on the philosophy of cooperatives. However, this type of shop offers most of the goods identical to conventional retail and does not seek to change the usual market-oriented relationship between producers and consumers.

^4^It should be stressed again that the shares of different types of direct purchases are shown in per cent of one fifth of the population which participates in this type of shopping. This means that, apart from direct purchase at a farm or farm’s shop, these types of shopping are marginal among the general population.

^5^Potential inclusion of this small subgroup into food growers would not change meaning of any of the analysis and conclusions in this study.

^6^For more details about the relationship of FSP in Eastern and Western Europe, different interpretations of very similar activities and the importance of knowledge production see, e.g., Jehlička et al. (74), Daněk et al. (50) or Jehlička (75).

^7^Simplified weights inspired by OECD equivalence scale (76) were used: first household member = 1, every other member = 0.7.

^8^We are aware of the complexity of the whole rural–urban categorization and other possible thresholds (e.g., 2,000 or 3,000).

^9^Given the effect of living in a family house on the participation in FSP and the overrepresentation of such types of houses in our sample, the exact share of 51.5% of food growing households might be slightly higher than in reality. However, it is in agreement with a previous recent study by Hanzlová (58).
