# Supplementary material for: An insight into market and non-market alternative food networks in Czechia during Covid-19 and beyond
Source: Front Nutr. 2024 Feb 6;11:1327308. doi: 10.3389/fnut.2024.1327308 (PMC10876859; doi:10.3389/fnut.2024.1327308)
Supplement: Supplementary file 1 [file Data_Sheet_1.docx]

**Supplement**

This supplement includes:

- A comparison of the sociodemographics of the survey sample and the Czech adult population (18+ years).
- The exact wording of the questions used in the survey (translation from Czech language). The questions are ordered as they appear in the article. This supplement does not include all questions from the questionnaire. For more details on food consumption and food habits see Millard et al. (2022) (ref. 5 in the Reference list) which presents an overview of a questionnaire study preceding our research.
- Frequencies of the answers to the questions.

**Table 1 Sociodemographic characteristics of respondents and the Czech adult population**

|  | Sample of respondents | Czech adult population |
| --- | --- | --- |
| **Sex** |  |  |
| Male | 48.9% | 48.7% |
| Female | 51.1% | 51.3% |
| **Age groups** |  |  |
| 18-29 | 15.5% | 16% |
| 30-39 | 18.6% | 17.8% |
| 40-49 | 21.2% | 19,4% |
| 50-59 | 16.1% | 15.2% |
| 60-69 | 15.5% | 16% |
| 70-79 | 13 | 15.6% |
| **Education** |  |  |
| Grammar | 10.9% | 11.3% |
| Lower middle | 34.6% | 35.1% |
| Upper middle | 36.7% | 35.9% |
| University | 17.9% | 17.7% |
| **Municipality size** |  |  |
| < 1,000 | 16.9% | 16.9% |
| 1,000–4,999 | 21.7% | 21.6% |
| 5,000–19,999 | 19.8% | 18.5% |
| 20,000–99,999 | 20.6% | 21.1% |
| 100,000+ | 21.0% | 21.9% |
| **Region** |  |  |
| Prague | 12.2% | 12.3% |
| Central Bohemian | 12.2% | 12.5% |
| South Bohemian | 6.2% | 6% |
| Pilsen | 5.4% | 5.5% |
| Karlovy Vary | 2.9% | 2.8% |
| Ústí nad Labem | 7.4% | 7.7% |
| Liberec | 4.9% | 4.1% |
| Hradec Králové | 5% | 5.2% |
| Pardubice | 4.9% | 4.9% |
| Vysočina | 4.9% | 4.8% |
| South Moravian | 10.7% | 11.2% |
| Olomouc | 6.2% | 6% |
| Zlín | 5.8% | 5.5% |
| Moravian-Silesian | 11.3% | 11.3% |

N=515. Note: Age categories in this table do not exactly correspond to the age categories used in the analysis in the paper as they were reduced into four categories only.

**Table 2 Food shopping**

| *% of Yes (shopping in the particular type of shop)* | What are the main ways of buying fresh fruit and vegetables for your household at the moment? | What are the main ways of buying other fresh food for your household at the moment (meat, fish, bakery, milk and dairy products)? |
| --- | --- | --- |
| Large retail (discount stores, supermarkets, hypermarkets) | 86.8% | 84.7% |
| Small food shops (greengrocers, bakeries, butchers, etc.) | 36.3% | 51.1% |
| Cooperative shops | 2.3% | 6.2% |
| Shops with organic, local, farmers’ or healthy food | 6.4% | 5.0% |
| Farmers’ markets (or other markets) | 18.8% | 11.1% |
| Directly from local growers or producers | 16.5% | 9.9% |
| Home delivery (online or phone order) | 6.8% | 6.0% |
| Other, explain… | 6.4% | 2.1% |

N=515. Both types of food were combined in the analysis as presented in the Tab. 1 in the article.

**Table 3 Direct purchase from local producers**

| *Multiple answers (Yes/No)* | If buying food from local growers or producers, can you specify which way of purchase do you choose? |
| --- | --- |
| Farm gate sale or farm shop | 71.7% |
| Pick-your-own, which crops… | 10.4% |
| Online order and pick up at the farm (e.g. box) | 12.3% |
| Online order and pick up at a selected place (e.g. a box) | 11.3% |
| Online order and home delivery (e.g. box) | 10.4% |
| Advertisements (e.g. on Facebook) and then we arrange the order and handover according to the situation | 17.0% |
| Member of Community Supported Agriculture (box according to seasonal harvest) | 0.9% |
| Other, explain… | 9.4% |

N=106. Only respondents who buy food directly from local growers or producers answered this question.

**Table 4 Food growing**

| *One option only* | When focusing on your own food production (gardening, animal husbandry), choose which answer best matches your household. |
| --- | --- |
| I produce my own food and want to produce more because of the recent experience with food during Covid-19 | 6.8% |
| I produce my own food, but the recent events during Covid-19 didn’t affect it | 44.7% |
| I don’t produce anything, but I'm thinking about it because of the experience during Covid-19 | 2.9% |
| I don’t produce anything, but I'm thinking about it, regardless of Covid-19 | 7.8% |
| I don’t produce anything, and I don’t even think about it | 35.9% |
| Other, explain… | 1.9% |

N=515. For the purpose of the analyses, a binary variable was created: growing (51.5%) and not growing (48.5%).

**Table 5 Where food is grown**

| *Multiple answers (Yes/No)* | Does your household have access to a place where fruit or vegetables can be grown? |
| --- | --- |
| Own garden at the house | 69.8% |
| Garden at weekend house | 18.9% |
| Growing in boxes/balcony | 17% |
| Relative’s garden | 13.6% |
| Allotment garden | 7.5% |
| Community garden | 0.8% |
| Other, explain… | 1.9% |

N=265. Only food growing respondents are included.

**Table 6 Amount of food produced**

| *One option only* | When thinking about the amount of food from your own production (fruits, vegetables, potatoes, eggs, etc.), to what extent do they cover your total food consumption compared to shopping? |
| --- | --- |
| Cover majority of consumption | 6.8% |
| It is an essential source of food, comparable with shopping | 9.8% |
| It is only a supplementary resource, majority is purchased | 42.6% |
| It is a negligible, rather seasonal, source | 40.4% |
| It has no importance | 0.4% |

N=265. Only food growing respondents answered this question.

**Table 7 Income change**

| *One option for each question* | Significantly decreased | Slightly decreased | No change | Slightly increased | Significantly increased |
| --- | --- | --- | --- | --- | --- |
| How has the economic situation of your household changed due to Covid-19? The household income has… | 6.6% | 22.3% | 59.4% | 10.5% | 1.2% |

N=515.

**Table 8 Food anxiety and shortages**

| *One option for each question* | Often | Sometimes | Never |
| --- | --- | --- | --- |
| Are you or someone else in your household worried about a lack of food that would not meet dietary needs (including cases of specific dietary needs), e.g. for financial reasons or due to supply failure? | 4.9% | 30.9% | 64.3% |
| Does it happen that there is sometimes not enough food in your household due to a lack of finance to buy it? | 2.7% | 15.0% | 82.3% |

N=515.

**Table 9 Food donations**

| *One option for each type of donation* | If you use any of the following forms of food donations, how important a source of food are they for your household? | | | | |
| --- | --- | --- | --- | --- | --- |
|  | They cover most of the consumption | It is an essential source of food | It is only a supplementary resource | It is a negligible resource | We don’t use them |
| Food banks | 0.6% | 1.0% | 2.3% | 0.8% | 95.3% |
| Food from charities | 0.2% | 0.6% | 2.3% | 0.8% | 96.1% |
| Various applications from restaurants (shops) | 0.4% | 1.0% | 3.1% | 4.1% | 91.5% |
| Gift from relatives | 0.8% | 2.7% | 10.3% | 21.4% | 64.9% |
| Gift from friends/neighbours | 0.2% | 0.6% | 5.0% | 15.7% | 78.4% |

N=515.

**Table 10 Changes in fresh fruit and vegetables shopping**

|  | How does the frequency of purchase in this place differ compared to the period before the Covid-19 pandemic? I shop there: | | | | | |
| --- | --- | --- | --- | --- | --- | --- |
|  | Much less | Little less | No change | Little more | Much more | Missing (doesn’t shop there) |
| Large retail (discount stores, supermarkets, hypermarkets) | 4.9% | 14% | 61.7% | 4.1% | 2.1% | 13.2% |
| Small food shops (greengrocers, bakeries, butchers, etc.) | 1.4% | 4.7% | 23.5% | 5.4% | 1.4% | 63.7% |
| Cooperative shops | 0% | .4% | 1.2% | .4% | .4% | 97.7 |
| Shops with organic, local, farmers’ or healthy food | .6% | 1.0% | 3.5% | 1.0% | 0.4% | 93.6% |
| Farmers’ markets (or other markets) | 1.7% | 4.3% | 8.9% | 2.7% | 1.2% | 81.2% |
| Directly from local growers or producers | 1.0% | 1.7% | 10.1% | 3.1% | 0.6% | 83.5% |
| Home delivery (online or phone order) | 0.4% | 0.2% | 2.5% | 1.7% | 1.9% | 93.2% |
| Other, explain… | 0% | 0% | 3.7% | 1.4% | 1.4% | 93.6% |

N=515.

**Table 11 Changes in other fresh food shopping (meat, fish, bakery, milk and dairy)**

|  | How does the frequency of purchase in this place differ compared to the period before the Covid-19 pandemic? I shop there: | | | | | |
| --- | --- | --- | --- | --- | --- | --- |
|  | Much less | Little less | No change | Little more | Much more | Missing (doesn’t shop there) |
| Large retail (discount stores, supermarkets, hypermarkets) | 3.7% | 13.8% | 62.1% | 3.9% | 1.2% | 15.3% |
| Small food shops (greengrocers, bakeries, butchers, etc.) | 1.2% | 5.2% | 35.5% | 7.4% | 1.7% | 48.9% |
| Cooperative shops | 0% | 0.6% | 3.9% | 1.4% | 0.4% | 93.8% |
| Shops with organic, local, farmers’ or healthy food | 0.4% | 0.6% | 3.1% | 0.8% | 0.2% | 95.0% |
| Farmers’ markets (or other markets) | 1.4% | 1.4% | 7.0% | 1.2% | 0.2% | 88.9% |
| Directly from local growers or producers | 0% | 0.6% | 6.2% | 2.3% | 0.8% | 90.1% |
| Home delivery (online or phone order) | 0.2% | 0.4% | 1.6% | 1.9% | 1.9% | 94% |
| Other, explain… | 0% | 0.2% | 1.4% | 0% | 0.6% | 97.9% |

N=515.

**Table 12 Changes due to Covid-19**

| *One option for each aspect* | Compared to the time before the Covid-19 pandemic, are your eating or shopping habits different now? | | | | |
| --- | --- | --- | --- | --- | --- |
|  | Much less | Slightly less | No change | Slightly more | Much more |
| Amount of food consumed | 2.1% | 15.0% | 72.6% | 8.7% | 1.6% |
| Amount of food from local growers or producers | 8.0% | 8.2% | 73.4% | 9.5% | 1.0% |
| Amount of food in organic quality | 15.5% | 6.4% | 69.5% | 7.6% | 1.0% |

N=515.

**Table 13 Food consumption decrease**

|  | Have you decreased consumption of the following type of food in your household due to financial reasons (compared do the situation before Covid-19)? |
| --- | --- |
|  | % of yes |
| Fresh fruit and vegetables | 9.5% |
| Fresh meat | 10.1% |
| Fresh fish | 16.5% |
| Bread and bakery | 2.7% |
| Milk and milk products | 4.5% |

N=515. The percentage does not reflect how often respondents consume the particular type of food, only the share of all respondents who reported some decrease of food consumption.

**Table 14 Frequency of food consumption**

| *One option for each type of food* | How often do you personally consume the following type of food? | | | | | |
| --- | --- | --- | --- | --- | --- | --- |
|  | Rarely or not at all (max. 1x a month) | 2-3x a month | 1x a week | 2-3x a week | 4-6x a week | Daily |
| Fresh fruit and vegetables | 1.6% | 2.9% | 9.5% | 23.9% | 27.0% | 35.1% |

N=515.
